# Identification of a Radiosensitivity Molecular Signature Induced by Enzalutamide in Hormone-sensitive and Hormone-resistant Prostate Cancer Cells

**DOI:** 10.1038/s41598-019-44991-w

**Published:** 2019-06-20

**Authors:** Maryam Ghashghaei, Tamim M. Niazi, Adriana Aguilar-Mahecha, Kathleen Oros Klein, Celia M. T. Greenwood, Mark Basik, Thierry M. Muanza

**Affiliations:** 1 Lady Davis Institute for Medical Research, Jewish General Hospital, Montreal, QC Canada; 2 Division of Experimental Medicine, McGill University, Montreal, QC Canada; 3 Department of Radiation Oncology, Jewish General Hospital, Montreal, QC Canada; 4 Department of Oncology, Jewish General Hospital, Montreal, QC Canada; 5 Segal Cancer Center, Lady Davis Institute of Research, Jewish General Hospital, McGill University, Montreal, QC Canada; 6 Department of Epidemiology, Biostatistics and Occupational Health, McGill University, Montreal, QC Canada; 7 Gerald Bronfman Department of Oncology, McGill University, Montreal, QC Canada; 8 Departments of Human Genetics, McGill University, Montreal, QC Canada; 9 Department of Surgery and Oncology, Jewish General Hospital, Montréal, QC Canada

**Keywords:** Prognostic markers, Prostate

## Abstract

Prostate cancer (PCa) is the most common cancer amongst men. A novel androgen receptor (AR) antagonist, enzalutamide (ENZA) has recently been demonstrated to enhance the effect of radiation (XRT) by impairing the DNA damage repair process. This study aimed to identify a radiosensitive gene signature induced by ENZA in the PCa cells and to elucidate the biological pathways which influence this radiosensitivity. We treated LNCaP (AR-positive, hormone-sensitive PCa cells) and C4-2 (AR-positive, hormone-resistant PCa cells) cells with ENZA alone and in combination with androgen deprivation therapy (ADT) and XRT. Using one-way ANOVA on the gene expression profiling, we observed significantly differentially expressed (DE) genes in inflammation-and metabolism-related genes in hormone-sensitive and hormone-resistant PCa cell lines respectively. Survival analysis in both the TCGA PRAD and GSE25136 datasets suggested an association between the expression of these genes and time to recurrence. These results indicated that ENZA alone or in combination with ADT enhanced the effect of XRT through immune and inflammation-related pathways in LNCaP cells and metabolic-related pathways in C4-2 cells. Kaplan–Meier analysis and Cox proportional hazard models showed that low expression of all the candidate genes except for PTPRN2 were associated with tumor progression and recurrence in a PCa cohort.

## Introduction

PCa is the most frequently diagnosed malignancy and is among the top three cancer-related deaths in American and Canadian men^1–3^. XRT therapy is one of the treatment options for locally advanced PCa. Unfortunately, 30–50% of patients undergoing XRT will experience a biochemical recurrence within 10 years^4^. Although the combination of XRT with ADT for high-risk localized PCa patients has demonstrated an improved overall survival rate, almost half of these patients develop a resistance to ADT. This resistance is caused by the incomplete blockade of AR-ligand signalling, AR amplifications, AR mutations, aberrant AR co-regulator activities, or AR splice variants expression with the development of castration-resistant tumors^5^. The AR is a type of nuclear receptor that is activated when bound by androgenic hormones. AR activation regulates the transcription of specific genes^6^. In 2012, ENZA, an AR antagonist, was approved by the FDA and Health Canada for the treatment of metastatic castration-resistant PCa (mCRPC)^7^. Castration-resistant PCa (CRPC) is defined by disease progression and a rise in serum prostate-specific antigen (PSA) levels despite ADT. CRPC represents the most aggressive type of PCa with a median survival rate of fewer than two years^8^. ENZA targets the AR signalling pathway at three key stages: (1) It blocks binding of androgens to the AR; (2) It prevents AR nuclear translocation within the nucleus; (3) It inhibits binding of activated AR to chromosomal DNA, which prevents transcription of target genes^9^. We have shown that ENZA enhances the effect of XRT through an impaired DNA damage repair process^10^. The aim of our research is to identify the radiosensitivity gene signature(s) induced by ENZA in PCa cells and to clarify the biological pathways that play important roles in the regulation of radiosensitivity.

From a clinical standpoint, the ability to predict tumor response to XRT therapy is a necessary avenue to improve treatment outcomes. Gene expression profiling is a major tool used to stratify which patients will benefit from radiosensitivity. These genomic data will also increase the understanding of the cellular mechanisms of intrinsic radioresistance in cancer. It is promising to integrate the biomarkers of radiosensitivity to develop personalized radiation therapy. Following radiation therapy, changes in gene expression have been detected in multiple cancer cell lines including PCa. Although p53, ATM mutations, and the loss of PTEN are associated with human PCa radiosensitivity, they do not accurately predict which individual tumor will eventually fail definitive radiation therapy^11^. Given the complexity of radiation-induced responses, comprehensive gene expression microarray analysis enables the identification of a wider range of genes and of signalling pathways involved in the response to radiation^12^. There are many factors that influence the transcriptional profile seen after exposure to irradiation such as genetic background, cell type, XRT dose, dose rate and time after XRT^13^. While radiation induces cell death through DNA damage, there are no clinically predictive markers available to indicate the likelihood of an effective treatment outcome^11^. Clinicopathologic factors and PSA levels both aid in decision making when selecting treatment for the individual patient, yet there is conflicting evidence as to the predictive and prognostic value of these markers^14^. In this study, in order to identify a common radiosensitivity gene signature and relevant biological pathways, we carried out gene expression profiling following treatment of LNCaP and C4-2 cell lines. We hypothesized that these gene signatures play a role in the radiosensitivity of PCa cell lines in different combined treatment modalities.

## Results

### Differentially expressed genes by one-way ANOVA in hormone-sensitive and hormone-resistant PCa cell lines

We have demonstrated that ENZA with or without ADT enhances the effect of XRT in both hormone-sensitive (LNCaP) and hormone-resistant (C4-2) PCa cell lines^10^. To identify radiosensitivity gene signatures and elucidate related signalling pathways, we performed gene expression analysis on the cells treated with XRT or ENZA ± ADT in combination with XRT. By performing a one-way ANOVA with contrasts, we determined the most significant *(p*-value < 0.0001) differentially expressed (DE) genes among the three experimental groups (ENZA + XRT vs. XRT, ADT + XRT vs. XRT, ENZA + ADT + XRT vs. XRT) in LNCaP (Table 1) and C4-2 (Table 2) cell lines, 13 and 11 genes were identified, respectively. The *NKX3-1* was the only common gene for all three comparisons and it appeared in both groups of LNCaP and C4-2 cells.

**Table 1:** The most significant DE from one-way ANOVA for each treatment condition (ENZA + XRT vs. XRT, ADT + XRT vs. XRT, ENZA + ADT + XRT vs. XRT) in LNCaP cell line.

Gene	ENZA + XRT	ADT + XRT	ENZA + ADT + XRT	AveExpr	*P*-Value	Adj.* P*-Value
*NKX3-1*	−0.8163	−1.085	−1.277	10.75	2.062e-06	0.05607
*LAT*	1.041	1.051	0.5554	5.165	1.124e-05	0.0882
*ZMIZ1*	−0.7282	−0.1722	−0.6085	8.726	1.251e-05	0.0882
*INSL6*	−0.7812	−0.83	−0.6041	3.513	1.314e-05	0.0882
*CDH10*	0.3829	1.172	0.8857	2.843	1.692e-05	0.0882
*SLC39A5*	0.02344	−0.3479	−0.9146	4.706	2.049e-05	0.0882
*PTPRN2*	0.6226	1.143	0.9196	4.765	2.271e-05	0.0882
*LRFN1*	−0.2878	−0.6021	−0.8624	6.478	3.009e-05	0.09189
*SPDEF*	−0.2894	−0.4062	−0.5095	10.28	3.042e-05	0.09189
*TEX44*	−0.4086	0.1263	−0.5077	5.724	4.161e-05	0.1131
*23069178*	−0.7734	−0.264	−0.6781	3.846	5.748e-05	0.1249
*PDE9A*	−0.7457	−0.5213	−0.9291	8.719	6.206e-05	0.1249
*MMEL1*	−0.3271	−0.7629	−0.262	6.291	6.437e-05	0.1249
*OSBPL10*	1.073	0.7663	1.023	5.868	6.79e-05	0.1249
*TC1800006649.hg.1*	−0.4451	0.354	−0.4623	3.297	6.891e-05	0.1249

XRT = Radiation, ENZA = Enzalutamide, ADT = Androgen deprivation therapy. DE = differentially expressed. *P*-values were corrected for multiple comparisons using FDR (B&H).

**Table 2:** The most significant DE from one-way ANOVA for each treatment condition (ENZA + XRT vs. XRT, ADT + XRT vs. XRT, ENZA + ADT + XRT vs. XRT) in C4-2 cell line.

Gene	ENZA + XRT	ADT + XRT	ENZA + ADT + XRT	Ave Expr	*P*-value	Adj. *P*-value
*CYP1A1*	0.5771	2.417	2.498	7.441	9.098e-08	0.002474
*TRIB2*	−1.052	−0.837	−1.336	5.552	6.11e-07	0.006839
*CYP1A2*	0.4795	3.093	2.894	7.047	7.546e-07	0.006839
*FBXL5*	0.01488	0.6239	0.5458	8.664	1.349e-06	0.009166
*CYP1B1*	−0.5141	0.6484	0.7994	5.068	1.762e-05	0.09561
*HTA2-neg-47421856_st*	0.1306	0.0377	2.3520	2.0598	2.11E-05	0.09561
*SEMA6A*	0.152	−0.5594	−0.6021	7.675	2.8e-05	0.1088
*TIPARP*	0.02464	0.7372	0.8546	7.523	4.115e-05	0.1354
*NKX3-1*	−0.6767	−0.497	−1.145	10.75	4.483e-05	0.1354
*PFKFB3*	−0.1228	−0.6118	−0.6407	6.928	6.377e-05	0.163
*PMEPA1*	−1.045	−0.4885	−1.259	8.374	6.594e-05	0.163
*C1orf116*	−0.8854	−0.03169	−0.6941	10.96	7.653e-05	0.1734

XRT = Radiation, ENZA = Enzalutamide, ADT = Androgen deprivation therapy. DE = differentially expressed. *P*-values were corrected for multiple comparisons using FDR (B&H).

Comparing the expression of genes observed in each experimental group to the gene expression of the non-radiated control group we identified two differently expressed genes. The results obtained from the comparative analysis of ENZA vs. control [CTR], ADT vs. CTR, ENZA + ADT vs. CTR in LNCaP are shown in Table 3, whereas the results obtained from the comparative analysis of the three C4-2 experimental groups are shown in Table 4. Using LNCaP cells we have identified 13 genes which were modulated following radiation, while *CYP1A1, CYP1A2*, and *FBXL5* were found to be deferentially expressed in both radiated and non-radiated conditions in C4-2 cells and, therefore, these genes are unlikely to be associated with regulation of radiation sensitivity.

**Table 3:** The most significant DE from one-way ANOVA for each treatment condition (ENZA vs. CTR, ADT vs. CTR, ENZA + ADT vs. CTR) in LNCaP cell line.

SYMBOL	ENZA	ADT	ENZA + ADT	AveExpr	*P*-Value	Adj. *P*-Value
*HTA2-pos 2978683_st*	−1.789	−1.74	−1.725	2.104	2.359e-09	6.414e-05
*CYP1A1*	0.6475	2.191	2.089	7.441	2.675e-06	0.0314
*HTA2-neg-47419193_st*	−0.5908	−0.9197	−0.8388	2.45	3.464e-06	0.0314
*CYP1A2*	−0.1485	2.119	2.198	7.047	4.064e-05	0.2762
*MUC7*	0.3477	−0.5006	0.1016	4.032	9.765e-05	0.4557

CTR = Control, ENZA = Enzalutamide, ADT = Androgen deprivation therapy. DE = differentially expressed. *P*-values were corrected for multiple comparisons using FDR (B&H).

**Table 4:** The most significant DE from one-way ANOVA for each treatment condition (ENZA vs. CTR, ADT vs. CTR, ENZA + ADT vs. CTR) in C4-2 cell line.

SYMBOL	ENZA	ADT	ENZA + ADT	AveExpr	*P*-Value	Adj. *P*-Value
*FBXL5*	0.1701	0.8815	0.7326	8.664	6.511e-09	0.000177
*CYP1A1*	0.6429	2.501	2.357	7.441	1.498e-07	0.001899
*CYP1A2*	0.4665	3.208	3.128	7.047	2.096e-07	0.001899
*CYP1B1*	−0.2779	0.8443	0.9887	5.068	1.133e-05	0.07704
*CCNG2*	0.0294	0.6596	0.6017	10.93	2.234e-05	0.1215
*TIPARP*	0.1094	0.7962	0.8448	7.523	5.932e-05	0.2588
*VSTM4*	0.8132	−0.06463	0.403	3.244	6.664e-05	0.2588

CTR = Control, ENZA = Enzalutamide, ADT = Androgen deprivation therapy. DE = differentially expressed. *P*-values were corrected for multiple comparisons using FDR (B&H).

Three genes (*LAT, PTPRN2*, and *PDE9A*) out of 13 genes identified to be differently expressed in the LNCaP had previously annotated functions in KEGG pathways. *LAT* as a gene involved in KEGG immune and inflammatory pathways, whereas *PTPRN2* and *PDE9A* were previously shown to be associated with type I diabetes mellitus pathway and purine metabolism, respectively.

In C4-2 cells, three genes (*CYP1A1, CYP1A2*, and *CYP1B1)* out of 11 genes identified to be differently expressed had previously annotated functions in the KEGG metabolic pathways as genes involved in tryptophan metabolism, steroid hormone biosynthesis, metabolism of xenobiotics by cytochrome P450, ovarian steroidogenesis, retinol metabolism, and in general as metabolic pathways regulators.

Comparisons of gene expression in the experimental groups to control non-radiated groups in LNCaP cells resulted in identification of differences in the expression of *MUC7* and *CCNG2* genes, which were annotated in KEGG as the genes involved in salivary secretion pathway, p53 signaling pathway, and foxo signaling pathways, respectively.

In brief, our results suggest that either ENZA alone, or in combination with ADT, may potentiate radiation response through immune and inflammation-related pathways in LNCaP cells and in metabolic-related pathways in C4-2 cells.

### Heatmap of differentially expressed genes based on the one-way ANOVA analysis of genes expressed in hormone-sensitive and hormone-resistant PCa cell lines

Hierarchical clustering based on the DE genes identified from one-way ANOVA results allowed to make a clear separation between genes modulated by XRT alone and ENZA with or without ADT in combination with XRT in LNCaP cells (Supplementary Fig. S1). While we had demonstrated a minor level of heterogeneity amongst the biological triplicates, the overall changes in gene expression induced by this treatment were consistent in all triplicates analysed allowing separation of the identified genes into two distinct clusters. In comparison to the XRT groups, the ENZA ± ADT in combination with the XRT group showed higher expression levels of *LAT* (involved in immune response pathways), *OSBPL10* and *PTPRN2* genes. Conversely, ENZA (with or without ADT) and XRT treated cells both showed lower expression levels of *NKX3-1*, *ZMIZ1*, *PDE9A*, and *SPDEF* genes which interact with AR in the same pathway.

The data generated from the gene expression analysis of C4-2 cells is illustrated as a gene expression heatmap of the DE genes graphically illustrating comparisons assessed using a one-way ANOVA. We demonstrated that there are two gene clusters (ADT + XRT with XRT and ENZA + XRT with ENZA + ADT + XRT). Decreased levels of expression of *C1 or f116, NKX3-1*, and *PMEPA1* genes were identified in ENZA ± ADT in combination with XRT condition compared to XRT ± ADT. Furthermore, ENZA + ADT treatment resulted an increase in the radiation response through downregulation of *PFKFB3, TRIB2*, and *SEMA6A* as well as the upregulation of *CYP1B1* and *TIPARP* (Supplementary Fig. S2) in C4-2 cells.

### Differentially expressed genes and KEGG annotation analysis with the addition of ENZA with or without ADT to XRT in hormone-sensitive and hormone-resistant PCa cell lines

The microarrays expression of ~27,000 genes after a cut off *p*-value < 0.0001 was applied. The most significant DE genes when the treatment included ENZA in addition to XRT relative to XRT alone were *LAT, ZMIZ1, INSL6,* and *OSBPL10* in LNCaP cells as well as *TRIB2, AAK1, FEZF2, OR4P4,* and *ADRB2* in C4-2 cells. Furthermore, we also identified genes that were significantly differentially expressed relative to XRT in ADT + XRT (Supplementary Tables S1 and 2) and ENZA + ADT + XRT in both LNCaP and C4-2 cells (Supplementary Tables S3 and 4). In the non-radiated group, the most significantly DE genes following treatment with ENZA, ADT, and ENZA + ADT in both LNCaP and C4-2 cellsare listed in Supplementary Tables 5–10.

Regarding the cells treated with ENZA, ADT and XRT, 4 out of the 16 genes (*NAPE-PLD, ZBTB16, LBH*, and *TANK*) are associated with known KEGG pathways in LNCaP cells. *TANK* is involved in KEGG immune-related pathways and *NAPE-PLD, ZBTB16, LBH* are known to be part of metabolic pathways. In addition, the 2 most upregulated genes (*DUSP19* and *OSBPL10*) have immune and metabolic functions. Analysis of gene expression in C4-2 cells revealed that the *CYP1A1* and *CYP1A2* are the most upregulated genes. These two genes were previously reported to be induced by ENZA treatment^15^. Our results demonstrated that their expression is upregulated by ENZA under no irradiated conditions as well.

### Venn diagram illustrating the overlap between the three conditions in hormone-sensitive and hormone-resistant PCa cell lines in comparison to radiation

The total number of genes in each condition was 27189. The Venn diagram demonstrated that the number of significantly different DE genes in LNCaP cells (166 genes: 80 up-regulated and 86 down-regulated) was much greater than C4-2 cells (80 genes: 45 up-regulated and 35 down-regulated). The Venn diagram also showed 17, 41, 60 genes in LNCaP cells and 21, 6, 23 genes in C4-2 cells that were deregulated (cut off *P*-value < 0.01) by ENZA + XRT, ADT + XRT, and ENZA + ADT + XRT treatments, respectively. Only 0.016% (2/118: *NKX3-1* (down-regulated), *INSL6* (down-regulated)) of the genes in LNCaP and 0.04% (2/50: *NOD1* (up-regulated), *TRIB2* (down-regulated)) of the genes in C4-2 cells were common among all three treatment conditions (Supplementary Fig. S3). Furthermore, the number of common genes between different treatment conditions in LNCaP and C4-2 cell lines is as follow: ENZA + XRT/ADT + XRT (LNCaP = 4, C4-2 = 0), ENZA + XRT/ENZA + ADT + XRT (LNCaP = 8, C4-2 = 5), and ADT + XRT/ENZA + ADT + XRT (LNCaP = 9, C4-2 = 7).

### Validation of microarray data by real-time quantitative RT-PCR

**Figure 1:**
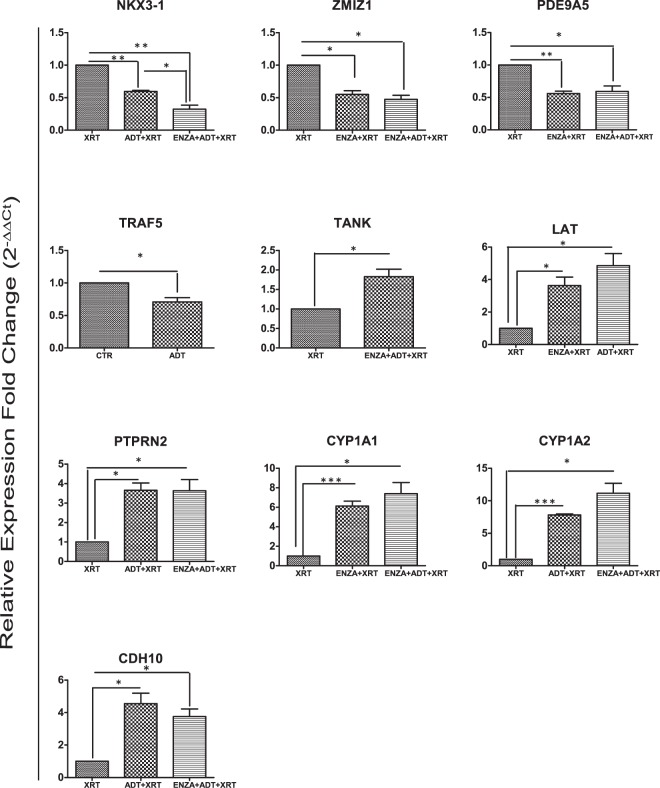
Validation by RT-qPCR of the microarray data. A set of up-regulated and down-regulated genes in LNCaP and C4-2 cells was analysed by RT-qPCR to validate the microarray data. All the RT-qPCR results were normalized to the expression level of GAPDH in each sample. The experiment was repeated three times and the results are presented as the mean ± SE. The level of significance in the statistical analysis is indicated as (*)*p*-value < 0.05, (**)*p*-value < 0.01, (***)*p*-value < 0.001 using two-tailed test. N = 3.

To confirm the microarray data, we selected 13 DE (the genes involved in immune-related pathways such as *LAT*^16^*, NKX3-1*^17^*, ZMIZ1*^18^*, TANK*^19^*, SPDEF*^20^, and *TRAF5*^21^, as well as the genes involved in metabolic-related pathways such as *CYP1A1*^22^*, CYP1A2,, PTPRN2*^23^*, OSBPL10*^24^*, KBTBD2*^25^*, SLC39A5*^26^, and *PDE9A*) genes from different treatment conditions and tested their expression using quantitative RT-PCR (qRT-PCR). The qRT-PCR analysis confirmed the differential expression of 9 out of 13 (69%) selected genes in the same direction predicted by the microarray analysis. These genes include *LAT, PTPRN2, NKX3-1, PDE9A, ZMIZ1, CYP1A1, CYP1A2, TANK* and *TRAF5* (Fig. 1).

### Analysis of the associations between candidate genes and risk of recurrence

**Figure 2:**
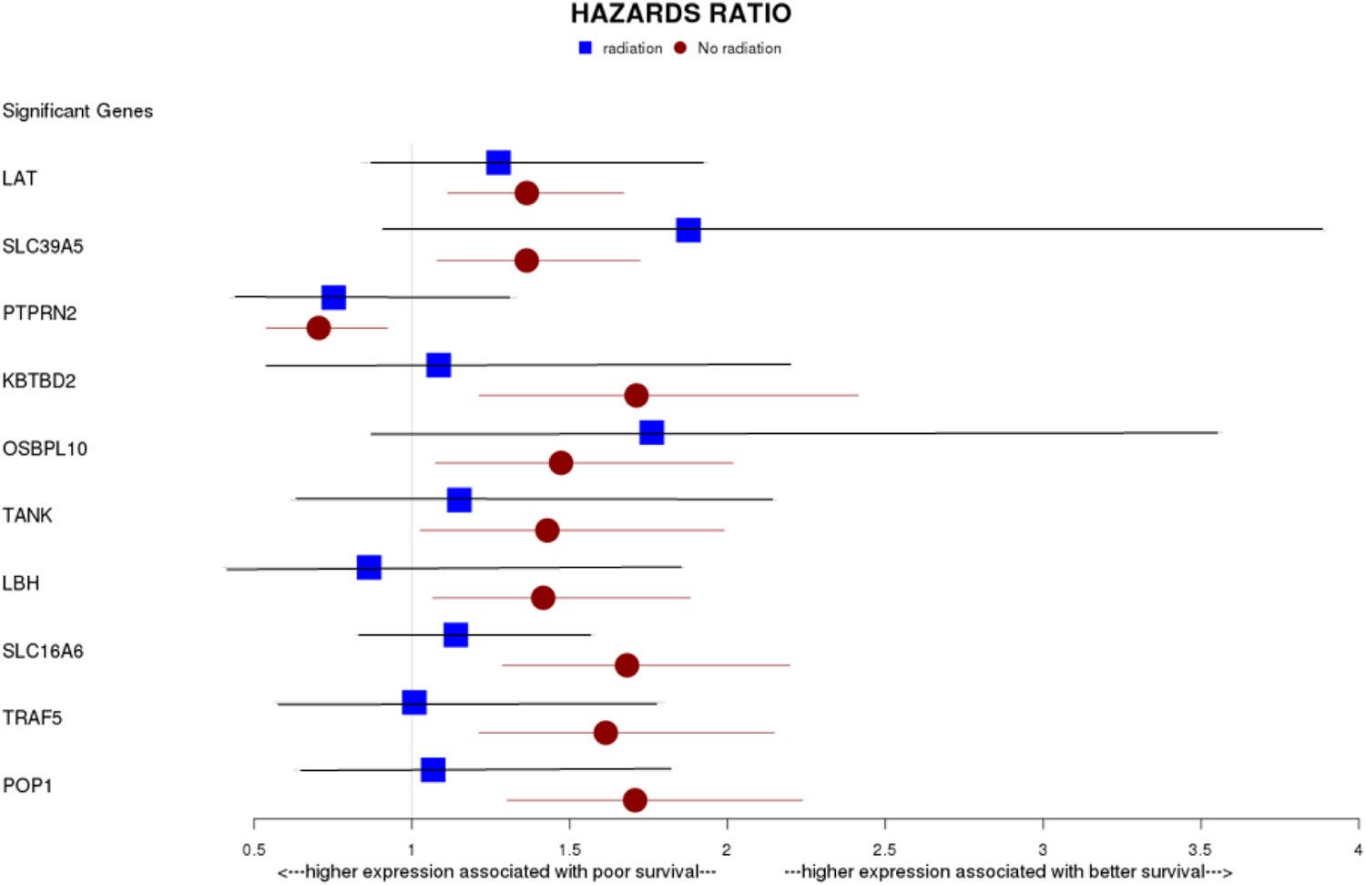
A forest plot of HR and 95% CI of the association between ten candidate genes and recurrence of PCa calculated from the univariate Cox regression analyses. Horizontal lines represent 95% CI. The blue squares and red circles correspond to the HR in radiated and non-radiated groups respectively. CI = confidence interval, HR = Hazard ratio.

**Figure 3:**
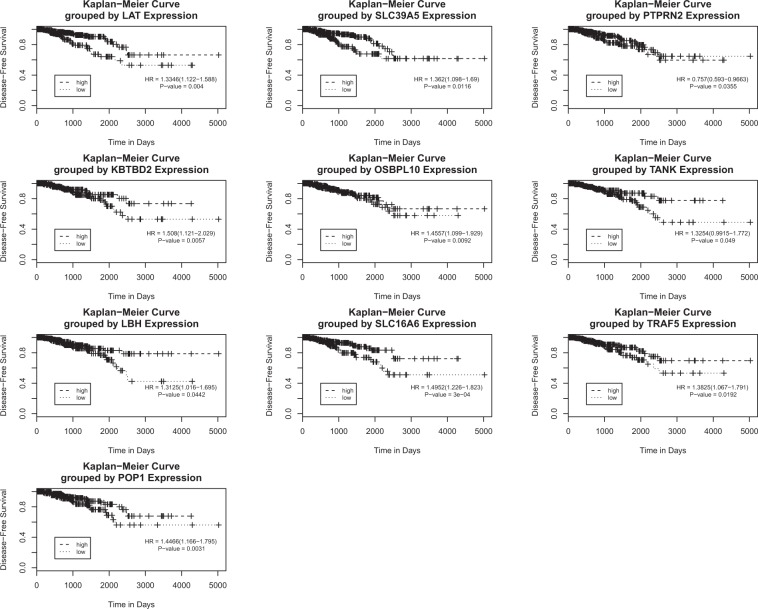
Kaplan–Meier disease free survival (DFS) curves. Kaplan-Meier curve compares the DFS in patients with high (above mean) and low (below mean) expression of the candidate genes, analysed from TCGA PRAD database (n = 438). The survival curves were compared using the log-rank test.

To evaluate the clinical significance of the candidate genes detected by one-way ANOVA analysis, we used The Cancer Genome Atlas Prostate Adenocarcinoma (TCGA PRAD) (n = 438) as our primary source of clinical information. In this database, 12.6% of patients received radiation therapy and 87.4% did not receive radiation therapy. We performed a univariate Cox proportional hazards survival model and found that the expression of a set of 10 genes was associated with time to recurrence (p < 0.05 without any correction for multiple testing). Figure 2 shows forest plot of the hazard ratio (HR) and confidence interval (CI) for each significant gene for patients treated with and without radiation. The radiation group did not reach the significance, perhaps due to low power (56 patients). The expression of the *KBTBD2* genes was associated with a HR for recurrence of 1.50 (P = 3.1 × 10^−4^) and for *PTPRN2* with an HR of 0.7 (P = 3.5 × 10^−2^) (Supplementary Table 11). The density plot showed the same distribution of all the genes in radiated and non-radiated groups (Supplementary Fig. S5). We also performed a multivariate Coxph model with ten significant genes and radiation status as a covariate. In the multivariate model, we found that only three of the genes that showed univariate significance (*SLC39A5, OSBPL10, and SLC16A6*) remained associated with survival which implies that only these three genes have independent effects on survival. Moreover, we identified that the interaction amongst the most significant genes (*SLC39A5, OSBPL10*, and *SLC16A6)* from the multivariate model with radiation status was not significant. Furthermore, the Kaplan-Meier curves showed a statistically significant difference between high-and low-gene expression groups in rates of disease-free survival (DFS) (Fig. 3, Supplementary Figs S6 and 7). DFS analysis revealed that patients bearing tumors with the lower expression of the genes (except *PTPRN2*) led to a significantly shorter time until recurrence (P < 0.05). Five-year DFS rate of all the candidate genes was shown in Supplementary Table S12. The 10-year survival rate was found to be higher in patients with a higher expression of these genes (Fig. 3).

### Expression levels of the ten candidate genes in human PCa patients

To examine the expression level of the candidate genes in human PCa tumors, we analysed RNA-Seq data from the TCGA PRAD dataset and microarray data from GSE25136 dataset. Both data sets contain log2-transformed FPKM (fragments per Kilobase million) values of all the genes. The expression of the top 10 candidate genes is summarized by two box plots from TCGA-PRAD and GSE25136 databases (Supplementary Fig. S8). The expression profile for the 10 genes selected based on the univariate Cox model results is different in the RNA sequencing data from the expression data derived from the Affymetrix array. The comparative analysis using TCGA database revealed *LAT* and *SLC39A5* mRNA had the lowest FPKM value among all the candidate genes, while *PTPRN2* expression had the highest of the other candidate genes with a median log2 FPKM value of about 6. The *PTPRN2* gene had a smaller effect size of recurrence compared to *LAT* and *SCLC39A5* genes. These results revealed that there is a reverse relationship between the HR of tumour recurrence and the expression level of *PTPRN2*, *LAT* and *SLC39A5* genes.

### The effects of the expression of candidate genes on recurrence

In the second PCa dataset (GSE25136), although time to recurrence was not available, a yes/no variable was available for recurrence. We tested whether the expression was different between these 2 datasets (TCGA PRAD, GSE25136). Linear regression analysis was used to test the effects of expression of the candidate genes on recurrence. According to the GSE25136 dataset, genes (including *AAK1, CYP1B1, NKX3-1, PTPRN2, TGF1,* and *TRIB2*) showed a different effect size of recurrence (*p* < 0.05 without any correction for multiple testing) (Table 5). The *PTPRN2* gene was common to both datasets with HR of 0.75 as well as −0.345 in the TCGA PRAD and GSE25136 datasets respectively. These results highlight the importance of the *PTPRN2* gene in evaluating the recurrence risk.

**Table 5:** Simple linear regression models showing the association of the variables with recurrence status using GSE25136.

Gene	Estimate	*P*-Value
*AAK1*	−0.4066	0.008166
*CYP1B1*	0.1428	0.04047
*NKX3-1*	0.9468	0.001266
*PTPRN2*	−0.345	0.01773
*TGIF1*	0.2587	0.01104
*TRIB2*	0.2482	0.005419

## Discussion

For decades, XRT has been used therapeutically to treat localized PCa. However, some patients treated with XRT experience biochemical recurrence within 10 years. Although ADT in combination with XRT increases overall survival in localized PCa, the disease progresses to CRPC due to the continued AR signalling pathway. We demonstrated that a profound AR inhibitor, ENZA, enhances the effect of XRT in both hormone-sensitive and hormone-resistant PCa disease^10^. Gene expression profiling is a valuable tool to elucidate the biomarkers of radiosensitivity in order to predict treatment response. These biomarkers would predict tumor response to radiotherapy and would identify subgroup(s) of patients that would not respond, thereby offering different treatment options^11^.

In this study, we demonstrate that ENZA alone or in combination with ADT increases radiosensitivity through immune and inflammation-related pathways in LNCaP cells, and through metabolic-related pathways in C4-2 cells. The Kaplan–Meier curve reveals that the low expression of the candidate genes in patients with PCa exhibit an earlier onset of tumor progression and recurrence compared with a the high expression of these genes in patients.

One-way ANOVA and KEGG annotations were performed to identify a radiosensitive gene signature induced by ENZA ± ADT in the PCa cells, and to clarify the biological pathways that play important roles in the regulation of radiosensitivity. The gene expression data were subjected to a one-way ANOVA analysis for each treatment condition (ENZA + XRT, ADT + XRT, ENZA + ADT + XRT).

We identified the most significant DE genes, *NKX3-1, ZMIZ1, SPDEF, PDE9A, LAT, PTPRN2*, and *OSBPL10* in LNCaP, and *CYP1A1* and *CYP1A2* in C4-2 cell lines.

*ZMIZ1* is an AR co-activator which enhances AR-mediated transcription^27,28^. The study by Peng and colleagues suggested that the inhibition of *ZMIZ1* reduced the growth of a human PCa cell line^29^. *ZMIZ1* is also a candidate oncogene in non-melanoma skin cancer. *ZMIZ1* is overexpressed in breast, ovarian, and colon cancers, and in human cutaneous squamous cell carcinoma^30^. It was reported previously that *ZMIZ1*, in collaboration with NOTCH1, induce T-cell acute lymphoblastic leukemia (T-ALL) in mice. *ZMIZ1* inhibition slows the growth of leukemic cells and increases their sensitivity to corticosteroids and NOTCH inhibitors^31^.

Zhang and colleagues reported that the androgen analog R1881 increased *NKX3.1* mRNA (a transcription factor expressed in epithelial cells of the prostate), and that the AR antagonist flutamide decreased *NKX3.1* mRNA in LNCaP cells^32^. *NKX3.1* was shown to inhibit estrogen receptor signaling in murine models of breast cancer^33^. *SPDEF*, a SAM pointed domain containing the ETS transcription factor, is up-regulated by androgen treatment and down-regulated by either the knockdown of AR or by treatment with bicalutamide^34,35^. Furthermore, the overexpression of *SPDEF* increases breast cancer progression^36^.

Dihydrotestosterone (DHT) increases the expression of *PDE9A*, a member of the cyclic nucleotide phosphodiesterase (PDE) family, in LNCaP cells^37^. *PDE9A* is also the main regulator of basal cyclic guanosine monophosphate (cGMP) levels in human breast cancer cells^38^. *PTPRN2*, an EMT-related (epithelial-mesenchymal transition) gene, is significantly higher in circulating tumor cells of CRPC patients than those of castration-sensitive patients^39^. Moreover, *PTPRN2* induces metastatic breast cancer cell migration through PI (4,5) P_2_‐dependent actin remodeling^40^. Dmitriev and colleagues identified *OSBPL10* as a biomarker of PCa. *OSBPL10* plays a key role in the maintenance of the body’s cholesterol balance^41^. Dobashi and colleagues reported *a* point mutation in the *OSBPL10* gene can be a prognostic indicator for diffuse large B-cell lymphoma^42^. *LAT*, a linker for the activation of T-cells, is a 36-Kd transmembrane protein that becomes rapidly tyrosine-phosphorylated by ZAP-70/Syk protein tyrosine kinases following activation of the T-cell antigen receptor (TCR) signal transduction pathway^16,43–45^. Sanada and colleagues reported DHT suppressed 3-methylcholanthrene (3MC)-induced transcription of CYP1 family (*CYP1A1, CYP1A2, CYP1B1*) in LNCaP cells^46^. Our data confirmed that the treatment of prostate cancer cells with ENZA enhances the effect of radiation through down-regulation of *NKX3-1*, *ZMIZ1*, *SPDEF* and *PDE9A* genes and up-regulation of *LAT*, *PTPRN2* and *OSBPL10* genes in LNCaP cells, as well as up-regulation of *CYP1B1* genes in C4-2 cells.

Furthermore, the KEGG annotation for the DE genes from one-way ANOVA revealed that overexpression of *LAT* following combination therapy induced radiosensitization of LNCaP cells though enhanced inflammation and immunity pathways. Such pathways include the Fc epsilon RI signalling pathway, Fc gamma R-mediated phagocytosis, Th1 and Th2 cell differentiation, NF-kappa B signalling pathway, T cell receptor signalling pathway and Th17 cell differentiation pathways. Androgen/AR exerts suppressive effects on T cell proliferation and modulates the balance of Th1 and Th2 responses^47–49^. In adaptive immunity, androgen/AR suppresses the development and activation of T and B cells^47^ and also inhibits Th17 differentiation^50–52^. Removal of such suppression by AR inhibition causes an enlarged thymus gland and extreme export of immature B cells^47^. The immunogenic modulation property of ENZA increases the sensitivity of PCa cells to T cell-mediated lysis, and this immunogenic modulation is dependent on AR^53,54^. ADT enhances Th1 differentiation of CD4 T cells and CD4-mediated immune responses by down-regulating the Ptpn1 gene, a direct target of AR, in PCa patients^51^. Moreover, ADT increases the infiltration of T cells into benign glands and tumor sites in human prostates^55^.

All these observations, taken together, strongly support the idea that AR inhibition by ENZA enhances the effect of radiation through immunity-related genes in hormone-sensitive PCa cells. In C4-2 cells, the most up-regulated genes (*CYP1A1*, *CYP1A2* and *CYP1B1*) are involved in steroid hormone biosynthesis and metabolism of xenobiotics by cytochrome P450 Weiss *et al*. also reported that ENZA induced the mRNA expression level of *CYP1A1* and *CYP1A2*^15^. These two genes were also found to be up-regulated in non-radiated controls, thus suggesting the modulation of expression of these two genes is not specific to the radiation response.

Importantly, we were able to validate most of the DE expressed genes (9 out of 13 genes) derived from microarray analysis by performing quantitative qRT-PCR. These DE genes are involved in immune pathways (e.g. *LAT, TANK*, and *TRAF5*) and metabolic pathways (e.g. *CYP1A1, CYP1A2*). Overall agreement of the qRT-PCR data with the microarray data was almost 69% (Fig. 1). The four (4/13) other genes showed a trend to significant differential expression with qRT-PCR (Supplementary Fig. S4).

As recurrence of localised PCa following treatment can lead to mCRPC, there is a critical need for the identification of reliable prognostic biomarkers to predict cancer recurrence following treatment of localized PCa. This led us to perform a univariate Cox proportional hazards survival model using the R package called ‘survival,’ and searching for association between expressions of each of the identified 55 candidate genes and the time to recurrence. We found a correlation between the expression of ten genes (10 out of 55 genes) and time to recurrence.

Furthermore, the effect size of recurrence in the non-radiated group compared to the radiated group was larger, which resulted in a stronger effect on DFS in these cohorts. This may be due to the small sample size in the radiated group (n = 56) versus the non-radiated group (n = 382). Furthermore, our density plot revealed a similar expression of all the genes in both groups. However, a multivariate Cox regression model of the entire cohorts, not only the under sized radiated subset, revealed that *SLC39A5, OSBPL10, SLC16A6* have a significant independent effect on survival. In conclusion, our results demonstrated that these three genes separately or together correlate with overall survival in patients with PCa.

Furthermore, by using the gene expression data and clinical data from patient cohorts (TCGA PRAD database) we have shown that the low expression of candidate genes (including *LAT*, *SLC39A5*, *KBTBD2*, *OSBPL10*, *LBH*, *SLC16A6*, *TANK*, *TRAF5* and, *POP1* genes) correlated with disease recurrence and poor patient prognosis. To confirm this, we separated patients into two groups according to high and low gene expression level and then subjected them to Kaplan-Meier analysis.

As shown in Fig. 3, the two groups of patients had significantly different times to recurrence. We found that low expressed genes (except *PTPRN2*) were associated with worse DFS as indicated by the presence of biochemical recurrence (rising PSA levels following local therapy) or radiological tumor recurrence/metastasis^56^. The 5-Year DFS was less in the low expression gene group than in the highly expressed gene group (Supplementary Table S12). Kaplan–Meier analysis demonstrated a complete separation of the curves between high and low expression of the *LAT*, *KBTBD2*, *TANK*, *LBH*, *SLC16A6* and *TRAF5* genes. Furthermore, the GSE25136 database revealed the effect of seven candidate genes including *AAK1*, *CYP1B1*, *NKX3-1*, *PTPRN2*, *TGF1* and *TRIB2* on the risk of recurrence. Among these candidate genes, *PTPRN2* was common between the two clinical datasets. As defined by the Gene Ontology Consortium, *PTPRN2* gene is involved in androgen receptor activity in PCa patients (Table 5). Lastly, we observed an inverse relationship between the expression of *PTPRN2*, *LAT* and *SLC39A5* genes and HR of tumour recurrence from the TCGA PRAD dataset. The significance of these weakly expressed genes and PCa recurrence warrants further investigation from the clinical trials biobank.

Although the present study revealed potential prognostic biomarkers for response to combined AR inhibitor and XRT therapy, it has a limitation. It would have been ideal to compare the effect of the candidate genes on recurrence between irradiated versus non-irradiated prostate cancer patients. Unfortunately, the sample size of irradiated patients (12.6%) was smaller than the non-irradiated, but the density plot analysis of each of the genes revealed similar expression in both groups.

The identification and validation of biomarkers for clinical applications remains an important issue for the purposes of improved diagnostics and therapeutic in many diseases, including PCa. Via gene expression profiles, we identified potential predictive biomarkers that correlate with clinical outcome. Currently, ENZA is an FDA approved drug for mCRPC. Our previously published data demonstrated its efficacy in modulating response to radiotherapy in both hormone-sensitive and hormone-resistant prostate cancer^10^. Those results and the radiosensitivity gene profile documented in this study provide well justified the pre-clinical rationale for clinical trials assessing the combination of ENZA with XRT at an early-stage of PCa than mCRPC. Our study documented strong evidence for the importance of simultaneous treatment with ENZA and XRT which might dramatically change the efficacy of XRT treatment and provide strong justification for assessing the predictive power of the identified markers in a clinical trial.

Overall, the gene signature described above predicted an enhanced radiation response to the combination therapy in our initial experiments^10^. Additionally, these genes are shown to be associated with an improvement in recurrence-free survival in the prostate cancer patients’ cohort of the TCGA. Therefore, these findings deserve further validation of predictive potential of identified markers in a clinical trial which would examine long term survival and efficacy of combined ENZA and XRT treatment in prostate cancer patients displaying identified molecular signature which would be enrolled into the study at an early-stage of PCa progression.

## Materials and Methods

### Reagents and PCa cell lines

Cell culture reagents were obtained from Gibco, Invitrogen (Burlington, Ontario, Canada). Fetal bovine serum (FBS) and charcoal stripped fetal bovine serum (CS-FBS) were obtained from Wisent Inc. (St-Bruno, Canada). ENZA was purchased from Selleckchem Com, (Cedarlane, and Paletta Court, Burlington, Ontario, Canada) and reconstituted in dimethyl sulfoxide (DMSO). LNCaP cells were obtained from ATCC (Manassas, VA) and C4-2 cells were provided by Dr. N. Zoubeidi (The Prostate Center, Vancouver General Hospital, University of British Columbia)^57^. The cells were cultured in RPMI-1640 supplemented with 10% (vol/vol) heat-inactivated fetal bovine serum (iFBS), 50 U/mL of penicillin, and 50 μg/mL of streptomycin. The cells were incubated at 37 °C in 95% air/5% CO_2_ and were tested for mycoplasma contamination using the mycoplasma PCR detection kit (Richmond, Canada) and found to be mycoplasma free. For hormone deficient treatment, ADT, we have used phenol red-free media supplemented with 10% charcoal dextran-treated serum (csFBS).

### Irradiation

LNCaP and C4-2 cell lines were irradiated to 4.0 Gy in a solid water equivalent phantom (sun nuclear corporation, Florida, USA) at the depth of 3 cm using 6 MV energy from a Varian Clinac EX machine **(**Palo Alto, California, United States). The source to phantom distance was 100 cm and the field size was 25 cm × 25 cm at the phantom surface. The dose rate of the machine was verified prior to cell irradiation using a calibrated ionization chamber, and the dose rate at the depth of the irradiated cells was verified independently using Eternal Beam Therapy films (EBT3 films, Ashland, Kentucky, USA) following the film dosimetry protocol established by Devic *et al*.^58^.

### RNA extraction

To study the differential gene expression, LNCaP and C4-2 cells were treated with ENZA (10 μmol/l) ± ADT 2 h before radiation (4 Gy), harvested 4 h later and the total RNA was isolated by the RNeasy Mini Kit (Qiagen, Valencia, CA) according to the manufacturer’s instructions. Experiments were repeated 3 times using newly plated cells for each replicate. RNA concentration and purity for each sample was verified with a Nanodrop ND-1000 spectrophotometer (Nanodrop, Rockland, DE, USA). The ratios of absorbance 260/280 nm and 260/230 nm were ~2 for all the samples, and the RNA concentration was ~500 ng/μL per sample. RNA integrity was determined with an Agilent 2100 Bioanalyzer (Agilent Technologies, Santa Clara, CA, USA). RNA Integrity Number (RIN) values close to 10 were obtained, indicative of high quality RNA samples. Pure RNA samples were stored at −80 °C until cDNA libraries were prepared.

### Expression microarray analysis

Sense-strand cDNA was synthesized from 100 ng of total RNA, and fragmentation and labelling were performed to produce ss-cDNA with the GeneChip reagent kit according to manufacturer’s instructions (Thermofisher Scientific-Affymetrix, Waltham, MA USA). After fragmentation and labelling, a 2.8 µg DNA target was hybridized on Clariom Sassay HT, human (Thermofisher Scientific -Affymetrix, Waltham, MA USA) and processed on a Gene Titan instrument (Thermofisher Scientific-Affymetrix, Waltham, MA USA) for hybridization -Wash-Scan automated workflow. For the analysis of the resulting data, several R packages from the bioconductor project (www.bioconductor.org) were used. Affymetrix gene expression microarray data was first background-corrected and normalized via the ‘rma’ method in the bioconductor package ‘oligo’. The RMA method includes background subtraction, normalization with the RMA algorithm and summarization using an approach called median-polish. The expression values are then transformed to the log2 scale. We performed a hierarchical clustering of the samples using the full panel of genes. The samples clustered well according to treatment conditions. Annotation for the Affymetrix Clariom S Assay HT for human was provided using the ‘clariomshumanhttranscriptcluster.db’ database provided by bioconductor. We applied two models for identifying genes that were differentially expressed. In model 1, we restricted the analysis to the radiated LNCaP cells.

We compared gene expression between three conditions: ENZA ± ADT in combination with XRT vs. XRT. In model 2 we repeated the same analyses in C4-2 cells. For all models we used the bioconductor package ‘limma’ to identify DE genes. A gene was regarded as being DE if *P-value* < 0.0001. The heatmap of genes from One-way ANOVA differential expression analyses was draw using the function heatmap.2 from the rpackage ‘gplots’. By default the clustering uses the ‘hclust’ function with the Euclidean distance. The microarray data have been submitted to the public functional genomics data repository Gene Expression Omnibus (GEO) under the accession number GSE126881.

### Quantile-quantile plot analysis

We mapped all the *p*-values on Q-Q plot (Supplementary Figs S9 and 10) and we compared what we have seen in our data (observed *p*-values) to what we expect (expected *p*-values)^59^. From our Q-Q plot, there is an elbow or an upward deviation of the observed *p* (black dots) values from the expected *p*-values (diagonal line) especially at the top right corner of the plot, so these genes are behaving differently than what we expect, that why we choose that threshold and that cut off *p*-value < 0.0001. Our sample size was very small, and there were ~27000 genes analyzed. *P*-values reported for each gene were corrected for multiple comparisons using Benjamini & Hochberg (B&H) false discovery rate (FDR)^60^. Due to the small sample size, the adjusted *p*-value was not significant for many of the genes identified, however we have adopted a highly stringent cut-off *p*-value (*p* < 0.0001) for significant changes.

### Real-time PCR

The DNase I (Qiagen, Valencia, CA) treated mRNA samples (1 µg) extracted from three independent biological replicates were reverse transcribed to cDNA using Super-Script III reverse transcriptase (Thermo Fisher scientific, Waltham, MA USA). Gene-specific primer pairs were designed using the NCBI Primer-BLAST tool (Supplementary Table S13). Amplification of the selected genes was performed using Applied Biosystems 7500 SYBR Green detection chemistry according to the manufacturer’s instructions. PCR amplification was preceded by incubation of the mixture for 60 s at 95 °C, and the amplification step consisted of 40–45 cycles. Denaturation was performed for 15 s at 95 °C, annealing was performed at 60 °C for 10 s, and extension was performed at 60 °C for 30 s, with fluorescence detection at 72 °C after each cycle. After the final cycle, a melting point analysis of all samples was performed within the range of 60 °C–95 °C with continuous fluorescence detection. qRT-PCR data were obtained from three independent experiments. The relative ratio of the threshold cycle (Ct) values between the endogenous controls (*GAPDH, ACTB*) and the specific gene was calculated for each sample. The validation procedure was performed with the same experimental design as for microarray analysis using the following genes (HGNC ID are in brackets): *LAT* (HGNC:18874), *OSBPL10* (HGNC:16395), *PTPRN2* (HGNC:9677), *SLC39A5* (HGNC:20502), *NKX3-1* (HGNC:7838), *SPDEF* (HGNC:17257), *ZMIZ1* (HGNC:16493), *PDE9A* (HGNC:8795), *CYP1A1* (HGNC:2595), *CYP1A2* (HGNC:2596), *TANK* (HGNC:11562), *TRAF5* (HGNC:12035), *KBTBD2* (HGNC:21751), *CDH10* (HGNC:1749), *ACTB* (HGNC: 132), *GAPDH* (HGNC:4141).

### Association with recurrence

Two publicly available databases, TCGA PRAD (https://portal.gdc.cancer.gov/) and GSE25136 (https://www.ncbi.nlm.nih.gov/geo/query/acc.cgi?acc=GSE25136), were used to assess the association between the expression of 10 candidate genes and time to recurrence. TCGA data for PRAD projects were downloaded from the TCGA portal maintained by GDC. Although the genes were identified in a microarray study, for the current project we use RNA sequencing data, specifically FPKM normalized expression values (fragments per kilobase million). Days to recurrence (RFS) and recurrence indicator (RFS_ind) were also obtained using the UCSC Zena Browser. Of the 498 samples, recurrence information was missing for 60 samples and therefore these samples were removed. In addition, data were downloaded from 79 prostate tumours from the GEO portal, project GSE25136. The data were normalized with Microarray Suite version 5.0 (MAS 5.0) with global scaling as the normalization method. A recurrence indicator variable was provided for each patient by the GSE25136 dataset.

Clinical and pathologic data (patient age, Gleason score, pathological T, pathological M, clinical-M, clinical-T, radiation therapy) for TCGA PRAD patients have been shown in Supplementary Table S14. We applied a univariate and multivariate Cox proportional hazards survival model and Kaplan-Meier plot, using the Coxph function in the R package ‘survival’. Time to recurrence was estimated with the Kaplan–Meier product limit estimator. Hazard ratios and *p*-values were estimated with univariate Cox proportion hazards models. Results were expressed as hazard ratios (HRs) with 95% confidence intervals (CIs). DFS curves were plotted according to the Kaplan-Meier method, and their *p*-values were calculated by the log-rank test for patients with the low (below mean) and high (above mean) expression of the genes. All differences were considered statistically significant at the level of *p* < 0.05. Statistical analyses were conducted in the R platform. The level of significance in the statistical analyses is indicated as *p < 0.05; **p < 0.01; ***p < 0.001; ****p < 0.0001.

## Supplementary information

Supplementary data

Supplementary Dataset S1

Supplementary Dataset S2

Supplementary Dataset S3

Supplementary Dataset S4
