# Supplementary material for: Identification of a Radiosensitivity Molecular Signature Induced by Enzalutamide in Hormone-sensitive and Hormone-resistant Prostate Cancer Cells
Source: Sci Rep. 2019 Jun 20;9:8838. doi: 10.1038/s41598-019-44991-w (PMC6586860; doi:10.1038/s41598-019-44991-w)
Supplement: Supplementary file 1 — Supplementary data [file 41598_2019_44991_MOESM1_ESM.pdf]

Supplementary Information and Dataset legends

Title: Identification of a Radiosensitivity Molecular Signature Induced by Enzalutamide in Hormone-sensitive and Hormone-resistant Prostate Cancer Cells

Authors list: Maryam Ghashghaei, Tamim M. Niazi, Adriana Aguilar-Mahecha,

Kathleen Oros Klein, Celia M.T. Greenwood, Mark Basik, Thierry Muanza

Supplementary Information

Heatmap of differentially expressed genes from one-way ANOVA in LNCaP cell line

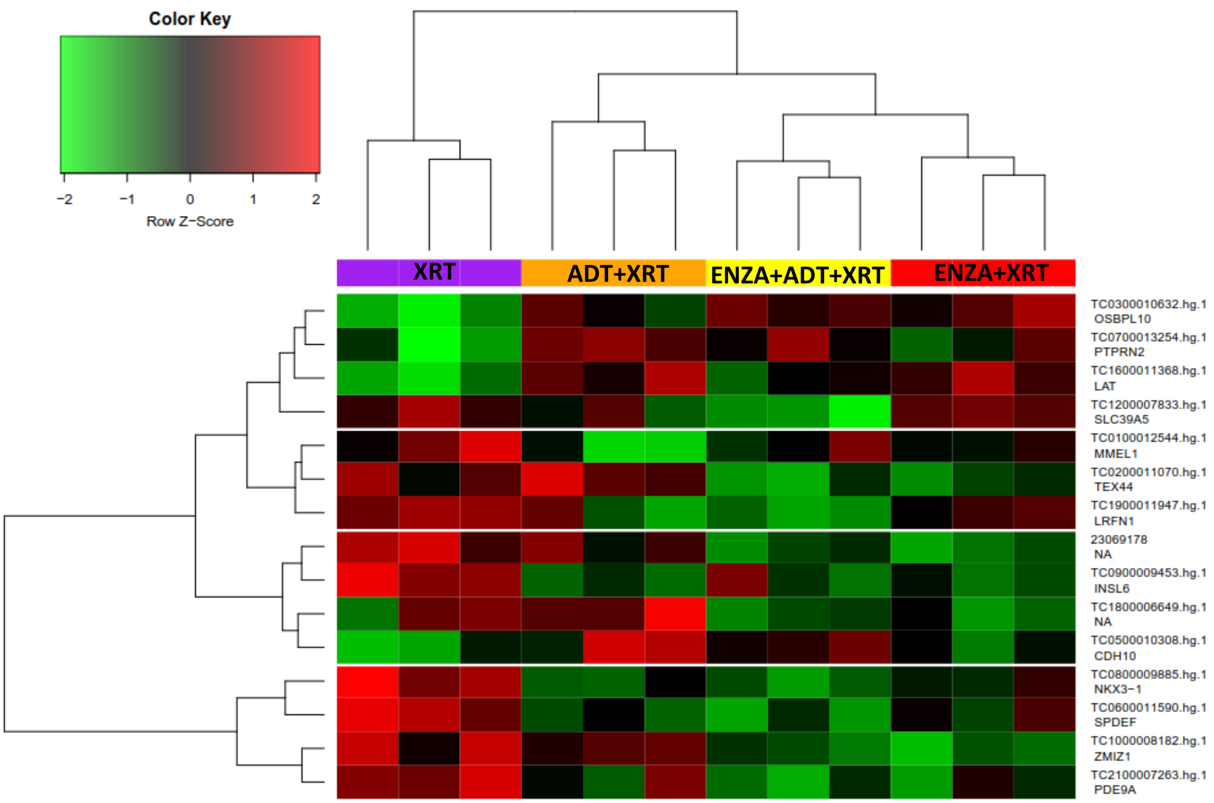

Supplementary Figure 1. Heatmap of DE genes from one-way ANOVA in LNCaP cell line. Purple, red, orange and yellow represent the four conditions, respectively: XRT, ENZA+XRT, ADT+XRT, and ENZA+ADT+XRT. XRT=Radiation, ENZA=Enzalutamide, ADT= Androgen deprivation therapy, DE= differentially expressed.

Heatmap of differentially expressed genes from one-way ANOVA in C4-2 cell line

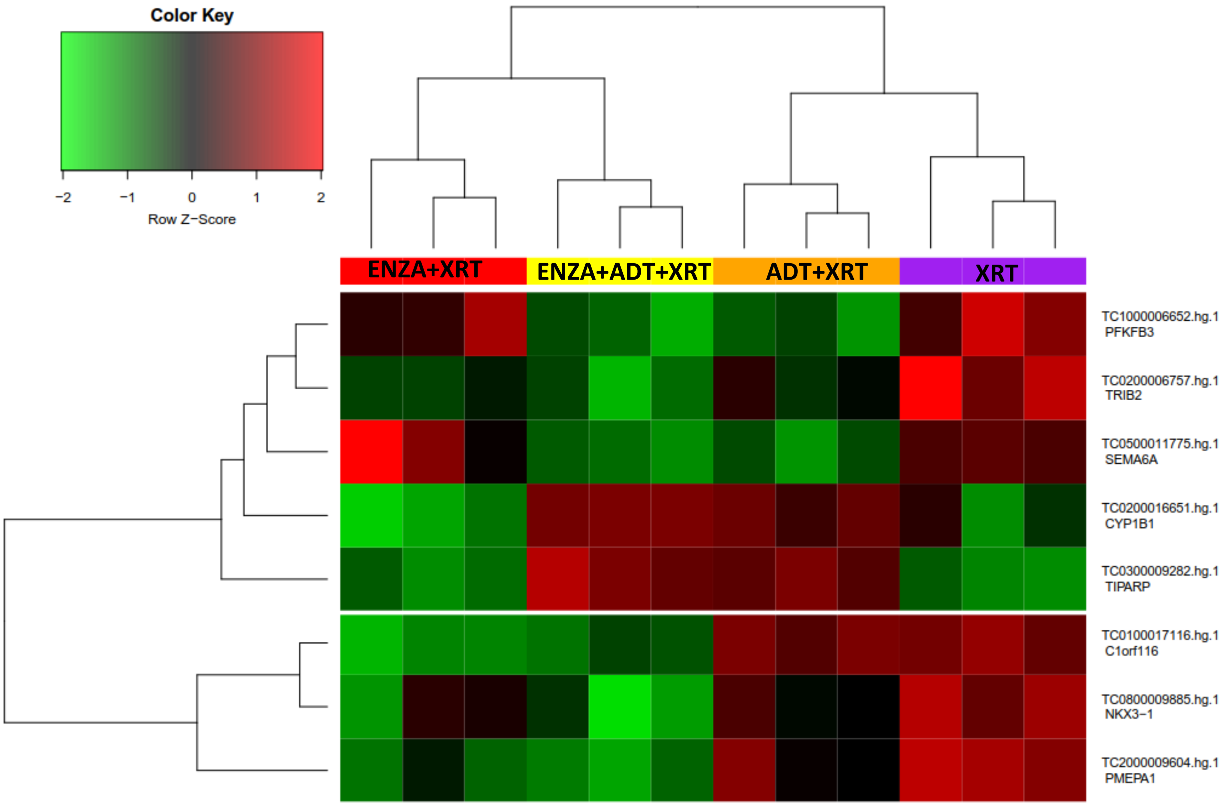

Supplementary Figure 2. Heat map of DE genes from one-way ANOVA in C4-2 cell line. Purple, red, orange and yellow represent the four conditions, respectively: XRT, ENZA+XRT, ADT+XRT, and ENZA+ADT+XRT. XRT=Radiation, ENZA=Enzalutamide, ADT= Androgen deprivation therapy, DE= differentially expressed.

The most significant DE genes between ADT+XRT vs. XRT in LNCaP cell line

Gene	log FC	P-Value	Adj. P-Value
PTPRN2	1.143	3.433e-06	0.03458
CDH10	1.172	3.947e-06	0.03458
MMEL1	-0.7629	4.743e-06	0.03458
INSL6	-0.83	5.398e-06	0.03458
NKX3-1	-1.085	7.445e-06	0.03458
LAT	1.051	7.632e-06	0.03458
KBTBD2	0.7891	2.587e-05	0.09145
CSDC2	-0.7017	2.691e-05	0.09145
**TC0700013330.hg.1**	0.7684	3.307e-05	0.09991
ZNF669	0.5804	5.724e-05	0.1556
MOCS1	-0.5972	9.925e-05	0.2366

Supplementary Table 1. The most significant DE genes between ADT+XRT vs. XRT in LNCaP cell line. XRT=Radiation, ADT= Androgen deprivation therapy, DE= differentially expressed. P-values were corrected for multiple comparisons using FDR (B&H).

The most significant DE genes between ADT+XRT vs. XRT in C4-2 cell line

Gene	Log FC	P-Value	Adj. P-Value
CYP1A1	2.417	7.505e-07	0.02041
CYP1A2	3.093	3.241e-06	0.04406
FBXL5	0.6239	9.19e-06	0.08329
CC2D2A	0.7147	8.507e-05	0.4707

Supplementary Table 2. The most significant DE genes between ADT+XRT vs. XRT in C4-2 cell line. XRT=Radiation, ADT= Androgen deprivation therapy, DE= differentially expressed. P-values were corrected for multiple comparisons using FDR (B&H).

The most significant DE genes between XRT+ ENZA+ADT vs. XRT in LNCaP cell line

Gene	Log FC	P-Value	Adj. P-Value
NKX3-1	-1.277	4.275e-07	0.01162
SPDEF	-0.5095	4.289e-06	0.04264
LRFN1	-0.8624	4.705e-06	0.04264
PDE9A	-0.9291	9.066e-06	0.06162
SLC39A5	-0.9146	1.509e-05	0.08203
NAPEPLD	0.6952	1.935e-05	0.08561
SLC25A37	-0.7242	2.204e-05	0.08561
TANK	0.5541	2.771e-05	0.09416
ZBTB16	-1.5	5.048e-05	0.1422
OSBPL10	1.023	5.229e-05	0.1422
FAM57A	-0.7497	5.967e-05	0.1475
23072083	0.9159	7.366e-05	0.1608
DUSP19	1.021	7.689e-05	0.1608
LBH	-0.6985	8.414e-05	0.1619
PTPRN2	0.9196	9.003e-05	0.1619
SLC16A6	-0.7354	9.786e-05	0.1619

Supplementary Table 3. **The most significant DE genes between XRT+ ENZA+ADT vs. XRT in LNCaP cell line.** XRT=Radiation, ENZA=Enzalutamide, ADT= Androgen deprivation therapy, DE= differentially expressed. P-values were corrected for multiple comparisons using FDR (B&H).

The most significant DE genes between XRT+ ENZA+ADT vs. XRT in C4-2 cell line

Gene	Log FC	P-Value	Adj. P-Value
TRIB2	-1.336	8.25e-08	0.002243
CYP1A1	2.498	4.065e-07	0.005527
NKX3-1	-1.145	3.036e-06	0.02752
CYP1A2	2.894	9.636e-06	0.0655
PMEPA1	-1.259	1.804e-05	0.09812
HTA2-neg-47421856_st	2.352	2.535e-05	0.1149
TGIF1	0.5261	4.256e-05	0.1653
FBXL5	0.5458	6.558e-05	0.2229

Supplementary Table 4. **The most significant DE genes between XRT+ ENZA+ADT vs. XRT in C4-2 cell line.** XRT=Radiation, ENZA=Enzalutamide, ADT= Androgen deprivation therapy, DE= differentially expressed. P-values were corrected for multiple comparisons using FDR (B&H).

The most significant DE genes between ENZA vs. CTR in LNCaP cell line

Gene	Log FC	P-Value	Adj. P-Value
HTA2-pos-2978683_st	-1.789	6.89e-09	0.0001873
FANCD2OS	-0.7816	4.601e-05	0.3744
LRP4	-0.5396	8.313e-05	0.3744
ZBTB16	-1.435	9.258e-05	0.3744
SYT8	0.8242	9.51e-05	0.3744

Supplementary Table 5. The most significant DE genes between ENZA vs. CTR in LNCaP cell line. CTR=Control, ENZA=Enzalutamide, DE= differentially expressed. P-values were corrected for multiple comparisons using FDR (B&H).

The most significant DE genes between ENZA vs. CTR in C4-2 cell line

Gene	Log FC	P-Value	Adj. P-Value
VSTM4	0.8132	7.962e-05	0.9998

Supplementary Table 6. The most significant DE genes between ENZA vs. CTR in C4-2 cell line. CTR=Control, ENZA=Enzalutamide, DE= differentially expressed. P-values were corrected for multiple comparisons using FDR (B&H).

The most significant DE genes between ADT vs. CTR in LNCaP cell line

Gene	Log FC	P-Value	Adj. P-Value
HTA2-pos-2978683_st	-1.74	1.276e-08	0.000347
HTA2-neg-47419193_st	-0.9197	1.146e-06	0.01558
CYP1A1	2.191	4.193e-06	0.038
OLFM4	0.9543	2.188e-05	0.1487
TC0600014205.hg.1	1.029	4.253e-05	0.2313
HTA2-pos-47421979_st	1.386	5.965e-05	0.242
ALPP	0.7721	7.238e-05	0.242
ZNF391	0.726	7.951e-05	0.242
TRAF5	-0.9546	8.295e-05	0.242
KCTD8	0.7943	8.901e-05	0.242

Supplementary Table 7. The most significant DE genes between ADT vs. CTR in LNCaP cell line. CTR=Control, ADT= Androgen deprivation therapy, DE= differentially expressed. P-values were corrected for multiple comparisons using FDR (B&H).

The most significant DE genes between ADT vs. CTR in C4-2 cell line

SYMBOL	Log FC	P-Value	Adj. P-Value
FBXL5	0.8815	1.356e-08	0.0003686
CYP1A1	2.501	3.978e-07	0.005408
CYP1A2	3.208	1.726e-06	0.01564
HTA2-neg-47422186_st	0.8041	2.205e-05	0.1499
ADAMTS3	-0.7052	5.788e-05	0.3147
SLC5A1	0.8682	7.697e-05	0.3488
CCNG2	0.6596	9.242e-05	0.359

Supplementary Table 8. The most significant DE genes between ADT vs. CTR in C4-2 cell line. CTR=Control, ADT= Androgen deprivation therapy, DE= differentially expressed. P-values were corrected for multiple comparisons using FDR (B&H).

The most significant DE genes between ENZA+ ADT vs. CTR in LNCaP cell line

SYMBOL	Log FC	P-Value	Adj. P-Value
HTA2-pos-2978683_st	-1.725	1.542e-08	0.0004193
HTA2-neg-47419193_st	-0.8388	5.567e-06	0.07568
CYP1A1	2.089	9.123e-06	0.08268
SYT8	0.8408	7.301e-05	0.4123
POP1	-0.6949	7.864e-05	0.4123
TC0900010900.hg.1	-1.004	9.325e-05	0.4123

Supplementary Table 9. The most significant DE genes between ENZA+ ADT vs. CTR in LNCaP cell line. CTR=Control, ENZA=Enzalutamide, ADT= Androgen deprivation therapy, DE= differentially expressed. P-values were corrected for multiple comparisons using FDR (B&H).

The most significant DE genes between ENZA+ADT vs. CTR in C4-2 cell line

SYMBOL	Log FC	P-Value	Adj. P-Value
FBXL5	0.7326	5.774e-07	0.0157
CYP1A1	2.357	1.191e-06	0.01618
CYP1A2	3.128	2.682e-06	0.02431

Supplementary Table 10. The most significant DE genes between ENZA+ADT vs. CTR in C4-2 cell line. CTR=Control, ENZA=Enzalutamide, ADT= Androgen deprivation therapy, DE= differentially expressed. P-values were corrected for multiple comparisons using FDR (B&H).

Venn diagrams of up-regulated and down-regulated genes in LNCaP and C4-2 cells

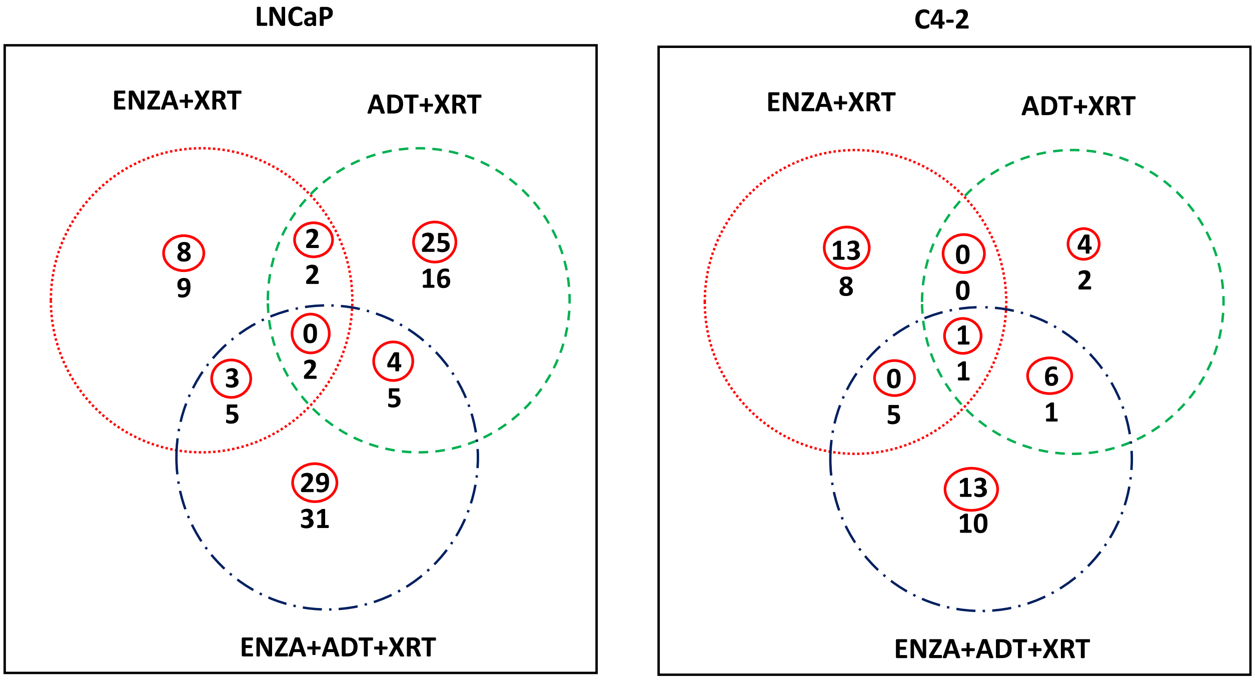

Supplementary Figure 3. Venn diagrams of up-regulated and down-regulated genes in LNCaP and C4-2 cells. The diagram shows deregulated genes (cutoff p- value<0.01) after treatment with ENZA+XRT, ADT+XRT and ENZA+ADT+XRT in LNCaP and C4-2 cell lines. The numbers in the oval represents the number of up-regulated genes in each treatment conditions and the numbers down the oval represents down-regulated gens. XRT=Radiation, ENZA=Enzalutamide, ADT= Androgen deprivation therapy. P-values were corrected for multiple comparisons using FDR.

Validation by RT-qPCR of the microarray data

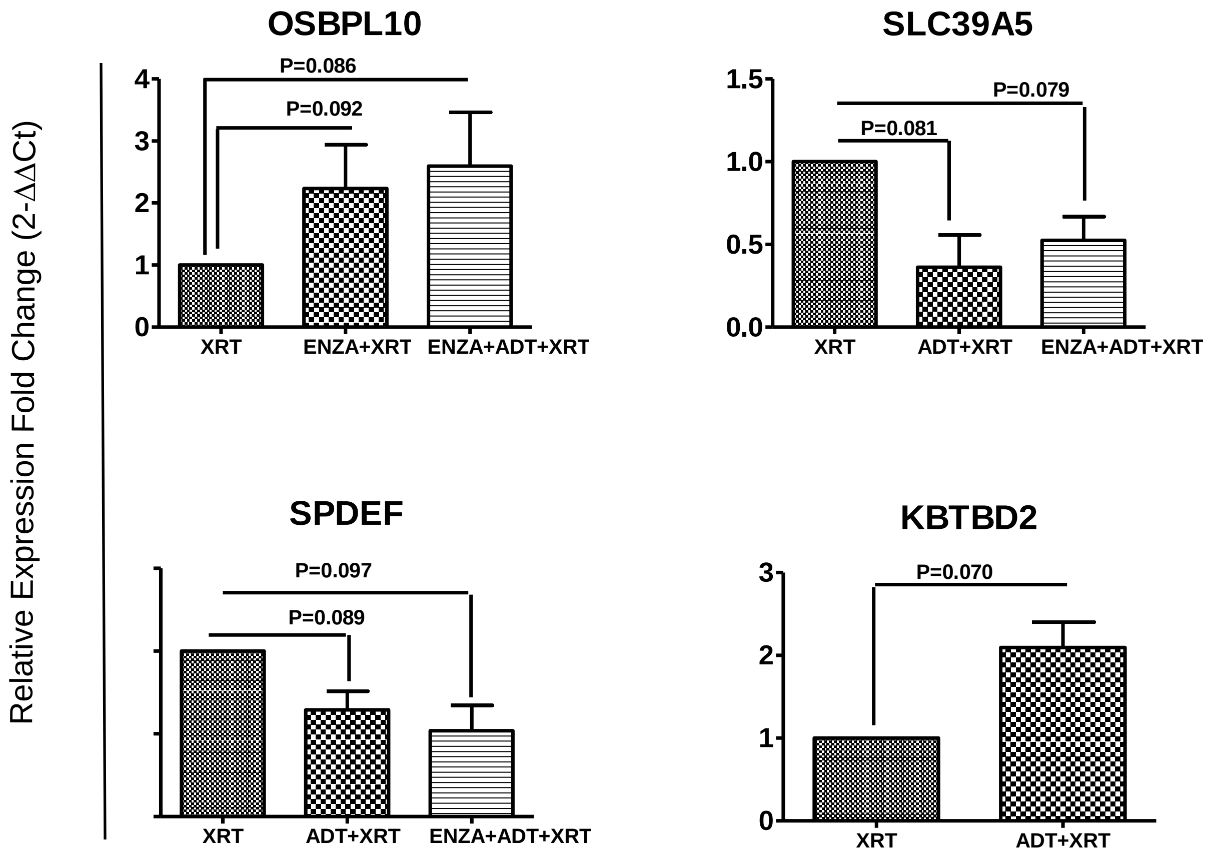

Supplementary Figure 4. Validation by RT-qPCR of the microarray data. A set of up-regulated and down-regulated genes in LNCaP cells was analyzed by RT-qPCR to validate the microarray data. All the RT-qPCR results were normalized to the expression level of GAPDH in each sample. Results are presented as the mean ± SE. The level of significance in the statistical analysis is indicated as (*) p-value <0.05, (**) p-value <0.01, (***) p-value <0.001 using two-tailed test.

List of hazards ratio and p-value for 10 significant candidate genes

Gene	P-value	HR	95% CI	HR (Radiation)	HR (No Radiation)
SLC16A6	0.000318	1.495	1.226-1.823	1.139	1.682
POP1	0.003125	1.447	1.166-1.795	1.067	1.707
LAT	0.00401	1.335	1.122-1.588	1.275	1.364
KBTBD2	0.005673	1.508	1.121-2.029	1.085	1.712
OSBPL10	0.009153	1.456	1.099-1.929	1.76	1.473
SLC39A5	0.01157	1.362	1.098-1.69	1.876	1.364
TRAF5	0.0192	1.383	1.067-1.791	1.008	1.614
PTPRN2	0.03551	0.757	0.593-0.9663	0.7521	0.7049
LBH	0.04417	1.313	1.016-1.695	0.8646	1.416
TANK	0.04896	1.325	0.9915-1.772	1.151	1.429

Supplementary Table 11. List of hazards ratio and p-value for 10 significant candidate genes, where significance was defined at p<0.05 without any correction for multiple testing. CI= Confidence interval. HR=Hazard ratio.

The density plot of 10 candidate genes using TCGA PRAD dataset

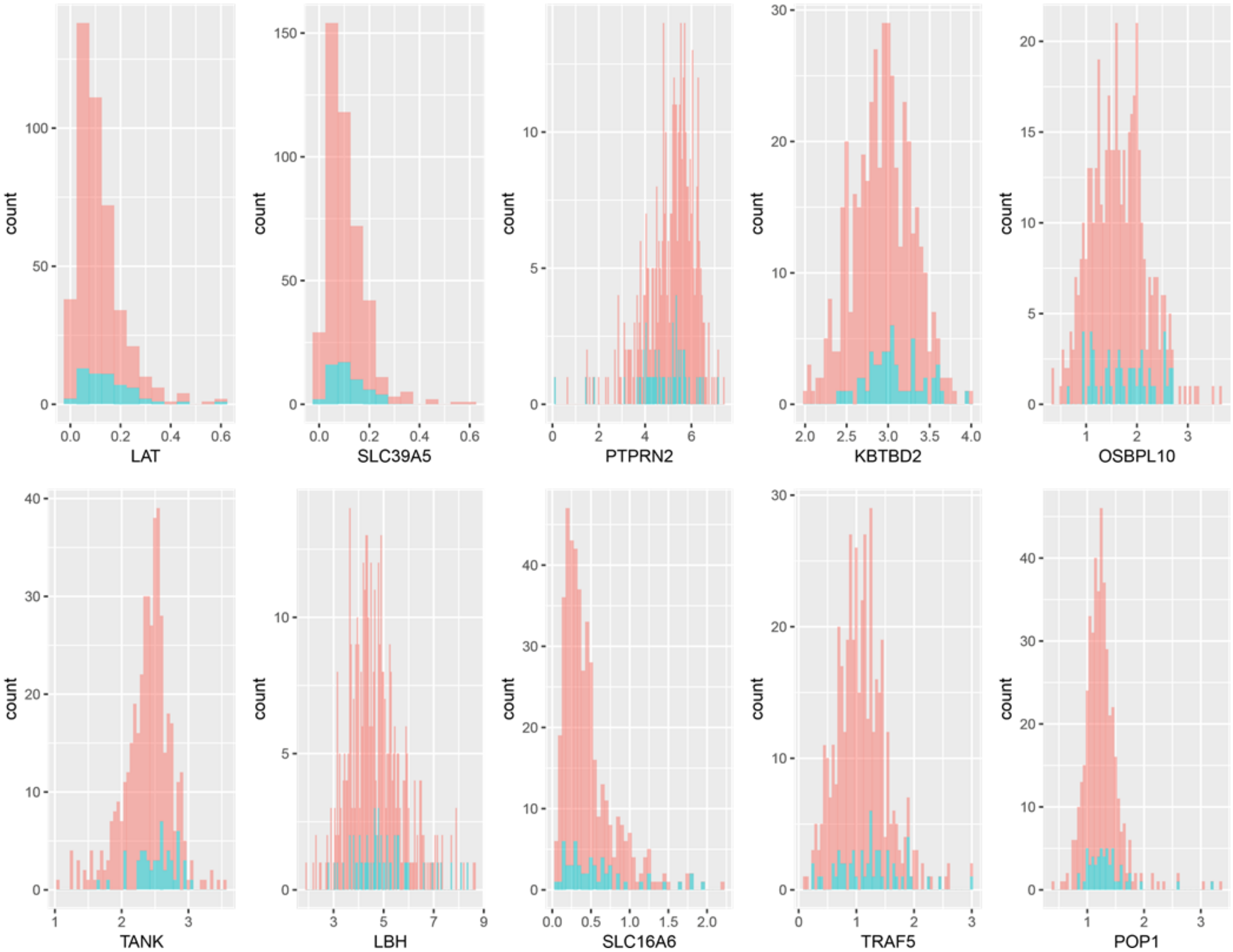

Supplementary Figure 5. The density plot of 10 candidate genes using TCGA PRAD dataset.

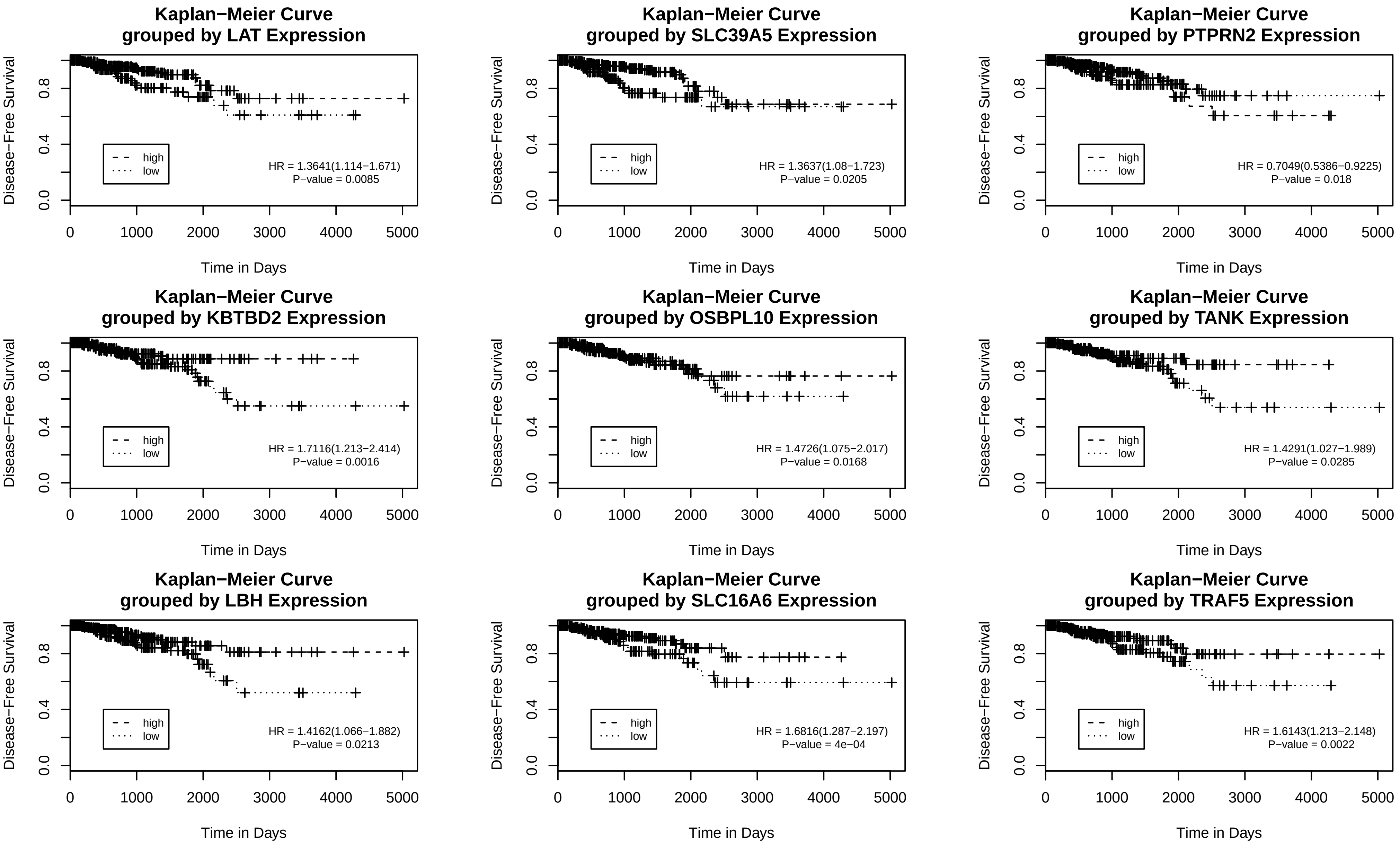

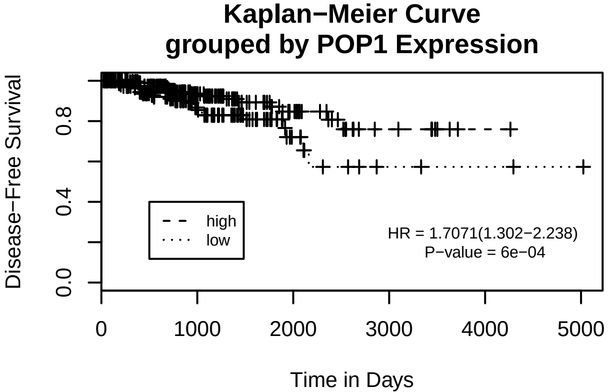

Supplementary Figure 6. Kaplan−Meier disease free survival (DFS) curves. Kaplan-Meier curve compares the DFS in patients with high (above mean) and low (below mean) expression of the candidate genes, analyzed from TCGA PRAD database in non -radiated group. The survival curves were compared using the log-rank test.

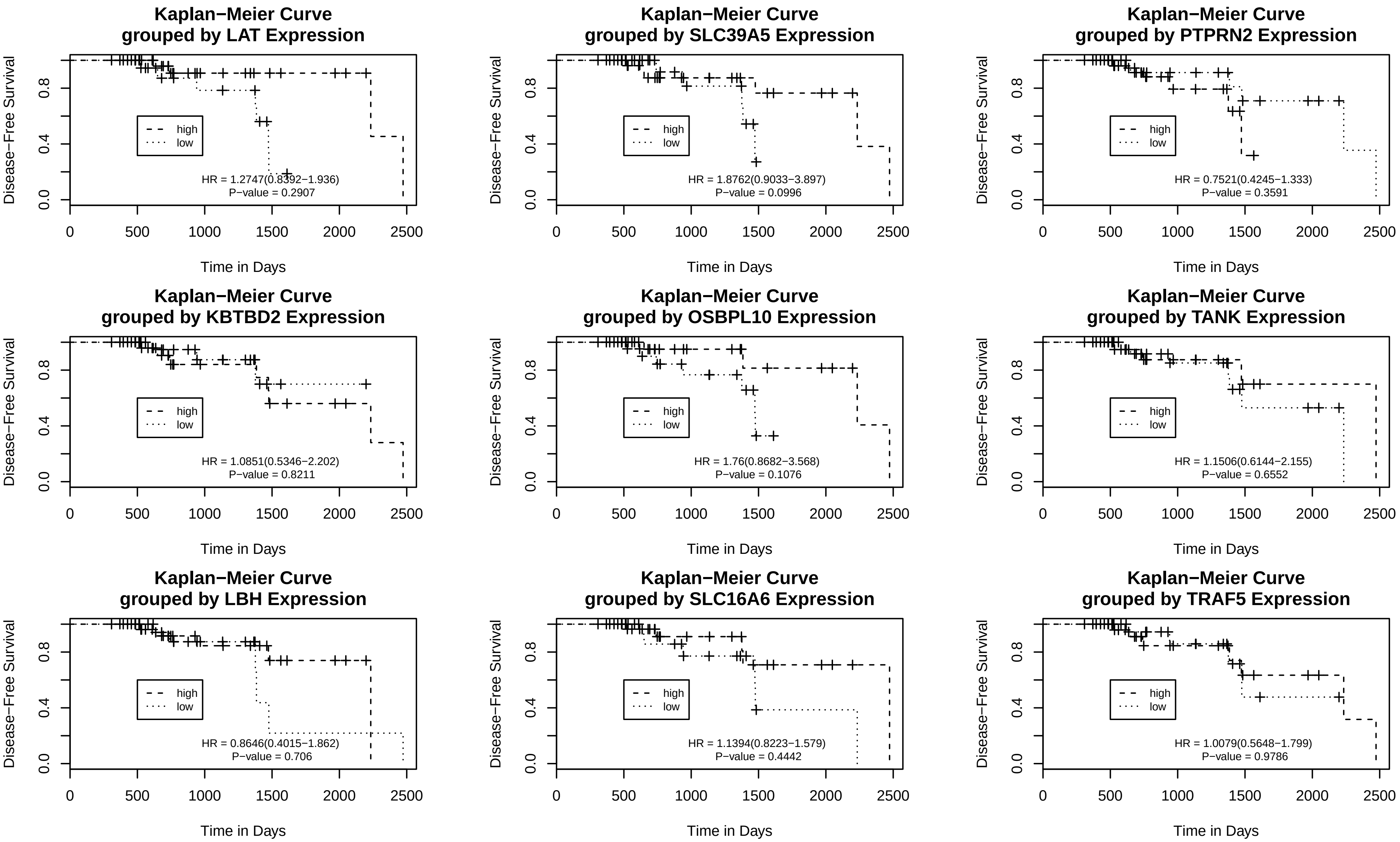

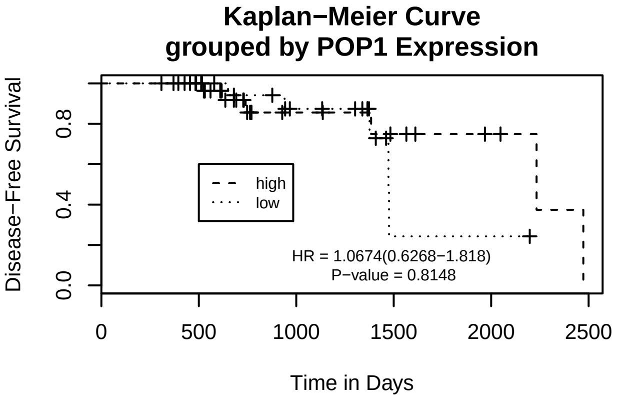

Supplementary Figure 7. Kaplan–Meier disease free survival (DFS) curves. Kaplan-Meier curve compares the DFS in patients with high (above mean) and low (below mean) expression of the candidate genes, analyzed from TCGA PRAD database in radiated group.The survival curves were compared using the log-rank test.

DFS rates of 5 -year for all the candidate genes

	**5-year DFS**	
	**Low gene expression**	**High gene expression**
**LAT**	64%	85%
**SLC39A5**	67%	85%
**PTPRN2**	72%	80%
**KBTBD2**	72%	85%
**TANK**	84%	88%
**OSBPL10**	72%	82%
**LBH**	72%	82%
**SLC16A6**	70%	82%
**TRAF5**	70%	82%
**POP1**	70%	85%

Supplementary Table 12. DFS rates of 5 -year for all the candidate genes. DFS= Disease free survival. Yrs=Year.

Box plot of FPKM values for selected candidate genes from TCGA-PRAD and GSE25136 datasets

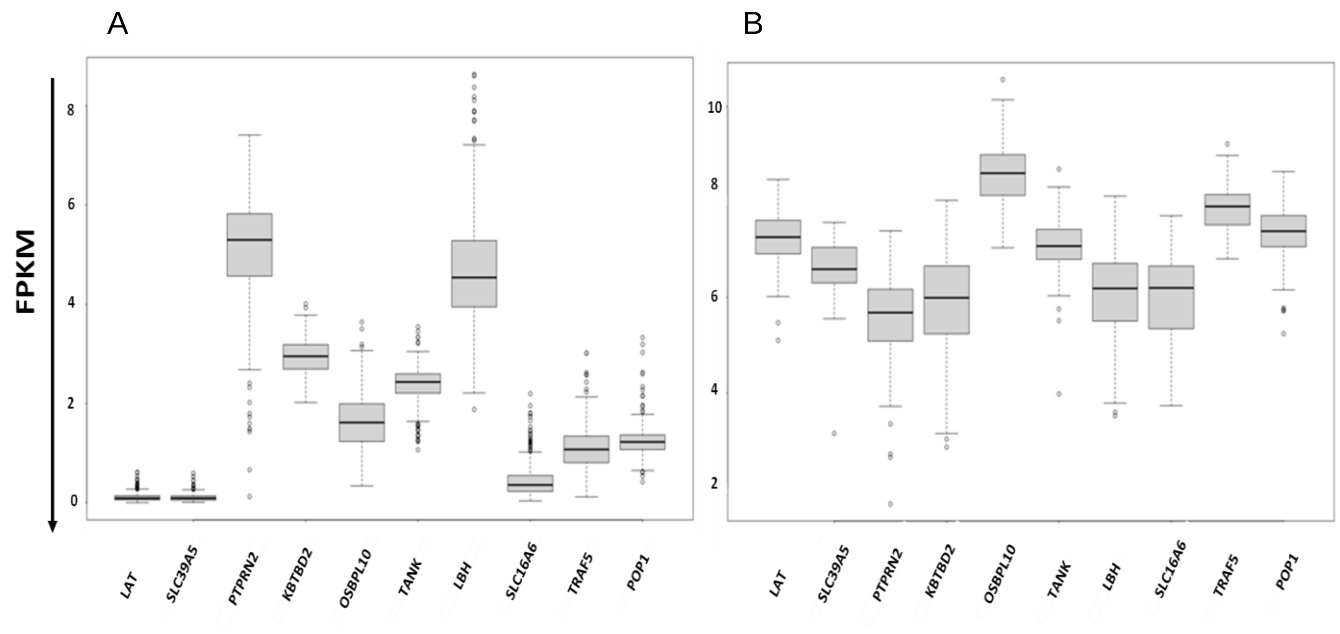

Supplementary Figure 8. Box plot of log2-transformed FPKM values for selected candidate genes from TCGA-PRAD (A) and GSE25136 (B) dataset. Box plots represent the differences in transcript levels of each candidate gene in two datasets. The median value is represented by the middle line in the boxes.

Primer sequences used in the qRT-PCR analysis

Gene	Sequence	TM
LAT-F	GAGCTACGAGAACGAGGGTG	64.5
LAT-R	CTGGTTCTGGGGGTAACGAC	64.5
OSBPL10-F	CATCGACACAACCACACTGC	62.4
OSBPL10-R	AAGTATACCCAGCCATCGCC	62.4
PTPRN2-F	GCCTGTGTGAACGATGGAGT	62.4
PTPRN2-R	TTCGGGAGGTCTGCAAGTTC	62.4
SLC39A5-F	GGAAGAGTCAAAGGCCCCTC	64.5
SLC39A5-R	CTGGGAGCCGTTCAGACAAT	62.4
NKX3-1-F	CAGAGACCGAGCCAGAAAGG	64.5
NKX3-1-R	CTGAGTGTGGGAGAAGGCAG	64.5
SPDEF-F	AAAGAGCGGACTTCACCTGG	62.4
SPDEF-R	CTGTGGGGCTTGAGTAGCAA	62.4
ZMIZ1-F	GGTCCCACCCAGGCGTAT	64.5
ZMIZ1-R	GAATAGGAAGGGACTGGGGC	64.5
TANK-F	GAGGTGCACCATCCATCACA	62.4
TANK-R	ACTAAGGATGCCGGGTCTCT	62.4

Gene	Sequence	TM
PDE9A-F	GGCCATCTACCTGGACATCG	64.5
PDH9A-R	TTGATGGCCACAGGTCTCAC	62.4
CDH10-F	GTGTGCTTGTGACAGCCAAG	62.4
CDH10-R	GAGGAGGATGGCGATCAAGG	64.5
ACTB-F	AGAGAGGCATCCTCACCCTG	60.69
ACTB-R	GATAGCACAGCCTGGATAGCA	59.65
TRAF5-F	AACCTGACCCCAATAGCAGC	62.4
TRAF5-R	TCAGTTAAGTCCACGGCCAC	62.4
GAPDH-F	GAGAAGGCTGGGGCTCATTT	62.4
GAPDH-R	AGTGATGGCATGGACTGTGG	62.4
KBTBD2-F	AGCCCCCAAGCAGAAAAAGT	60.4
KBTBD2-R	TGCGGACAAAAAGCATTGGG	60.4
CYP1A1-F	CACGGAGTTTCTTCTGGCCT	62.4
CYP1A1-R	CAATTCGGATCTGCAGCACG	62.4
CYP1A2-F	TGTCAGTGGCCAACGTCATT	60.4
CYP1A2-R	GAAGAAGTCCAGGGGGTTCC	64.5

Supplementary Table 13. Primer sequences used in the qRT-PCR analysis.

TM= Melting temperature, F=Forward, R=Reverse.

Quantile-quantile plots

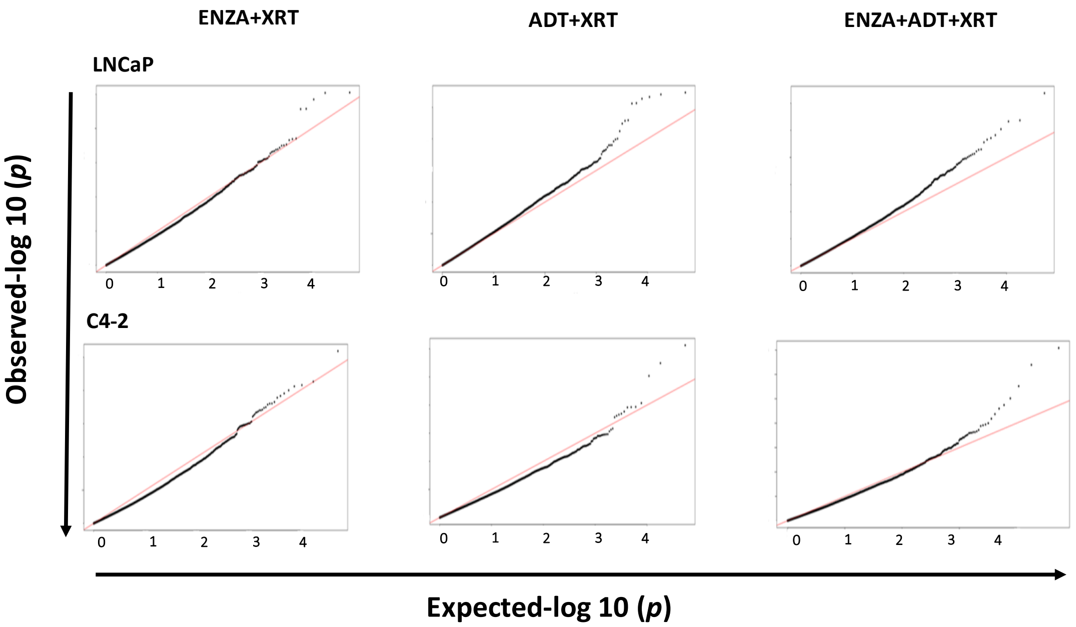

Supplementary Figure 9. Quantile-quantile plot of observed-log 10 (p) values vs. expected-log 10 (p) values in LNCaP and C4-2 cells in radiated groups (ENZA+XRT, ADT+XRT, ENZA+ADT+XRT). Diagonal line (expected p-values) indicates a p-value distribution. Upward deviations indicate p-values more significant than expected. XRT=Radiation, ENZA=Enzalutamide, ADT= Androgen deprivation therapy.

Quantile-quantile plots

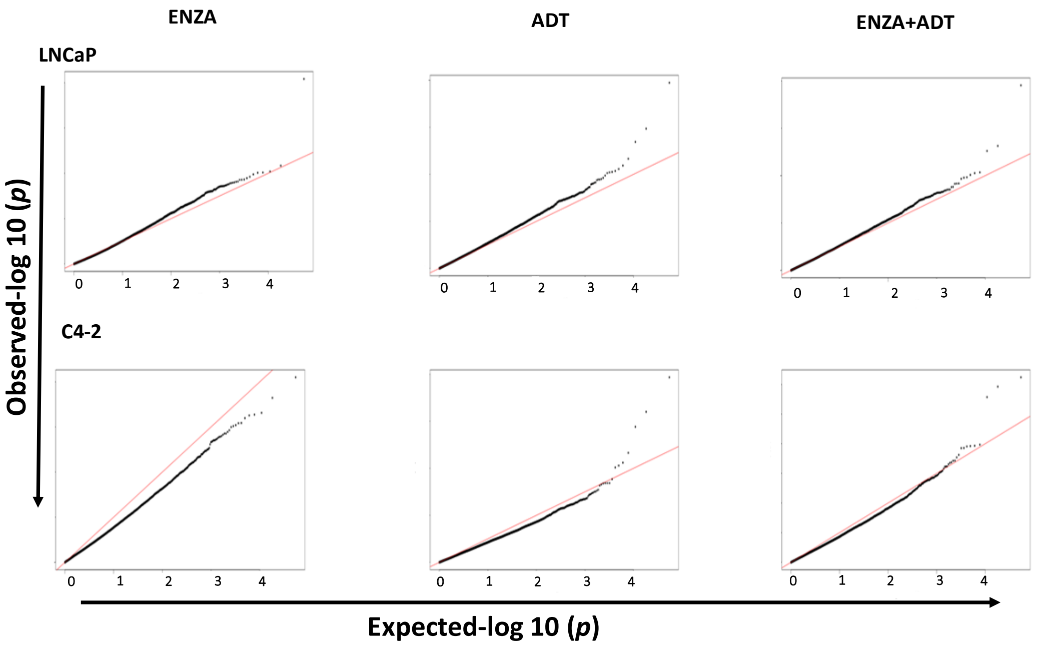

Supplementary Figure 10. Quantile-quantile plot of observed-log 10 (p) values vs. expected-log 10 (p) values in LNCaP and C4-2 cells in non-radiated groups (ENZA, ADT, ENZA+ADT). Diagonal line (expected p-values) indicates a p-value distribution. Upward deviations indicate p-values more significant than expected. CTR=Control, ENZA=Enzalutamide, ADT= Androgen deprivation therapy.

Pathological and clinical data for prostate cancer

	Patients (n=438)
**Age (Year)**	60 (41-80)
**GS**	
≤6	35 (7.98%)
=7	229 (52.24%)
≥8	170 (38.81%)
**Pathological T**	
T4	10 (2.27%)
T3a	145 (33.0%)
T3b	115 (26.3%)
T2a	10 (2.27%)
T2b	9 (1.95%)
T2c	150 (34.3%)
**Pathological N**	
N0	360 (82.2%)
N1	78 (17.8%)
**Biochemical Recurrence**	
Yes	56 (12.8%)
No	382 (87.2%)
**Clinical-M**	
M0	436 (99.5%)
M1a	1 (0.175%)
M1b	1 (0.175%)
M1c	1 (0.175%)
**Clinical-T**	
T4	2 (0.398%)
T3a	42 (9.54%)
T3b	17 (3.78%)
T2a	63 (14.3%)
T2b	63 (14.3%)
T2c	54 (12.3%)
T1a	1 (0.199%)
T1b	2 (0.398%)
T1c	185 (42.1%)
**Radiation Therapy**	
Yes	55 (12.6%)
No	383 (87.4%)

Supplementary Table 14. Pathological and clinical data for prostate cancer

Supplementary Datasets

Supplementary Dataset S1. Differential expression results for different treatment conditions (ENZA+XRT vs. XRT, ADT+XRT vs. XRT, ENZA+ADT+XRT vs. XRT) in LNCaP cell line. Genes are ordered by their P-values. ENZA=Enzalutamide, ADT=Androgen deprivation therapy, XRT=Radiation therapy.

Supplementary Dataset S2. Differential expression results for different treatment conditions (ENZA+XRT vs. XRT, ADT+XRT vs. XRT, ENZA+ADT+XRT vs. XRT) in C4-2 cell line. Genes are ordered by their P-values. ENZA=Enzalutamide, ADT=Androgen deprivation therapy, XRT=Radiation therapy.

Supplementary Dataset S3. Differential expression results for different treatment conditions (ENZA vs. CTR, ADT vs. CTR, ENZA+ADT vs. CTR) in LNCaP cell line. Genes are ordered by their P-values. ENZA=Enzalutamide, ADT=Androgen deprivation therapy, CTR=Control.

Supplementary Dataset S4. Differential expression results for different treatment conditions (ENZA vs. CTR, ADT vs. CTR, ENZA+ADT vs. CTR) in C4-2 cell line. Genes are ordered by their P-values. ENZA=Enzalutamide, ADT=Androgen deprivation therapy, CTR=Control.
